# Editorial: Brain modifications in response to stress: From cellular to circuit reorganization

**DOI:** 10.3389/fnsys.2022.1000279

**Published:** 2022-08-22

**Authors:** Arun Asok

**Affiliations:** Alien Therapeutics Inc., Philadelphia, PA, United States

**Keywords:** stress, fear and anxiety, posttraumatic stress disorder (PTSD), emotions, neural circuit

Stress is ubiquitous, conserved across species, and intimately penetrates our daily lives. Nearly a century ago, prior to and following World War I, Walter Canon defined how arousal and the broader sympatho-adrenal system directs our “fight or flight” response (cf. Cannon, 1922). Canon's work conceptualized how emotions, such as fear, are inextricably linked to stress. In the following years, Selye (1946), himself a Nobel Prize contender in 1949, parceled out psychological and physiological aspects of stress—defining both positive “eustress” and negative “distress” states. Selye's studies on how external stressors impact organs such as the adrenal were paralleled by several elegant studies in the 1950s and 1960s by Nobel laureates Roger Guillemin and Andrew Schally. Guillemin and Schally's work penetrated the cloaked vale of the brain by identifying how a region near the hypothalamus releases specific peptides, such as corticotropic-releasing or adrenocorticotropic hormone (CRH or ACTH; cf. Saffran and Schally, 1955; Guillemin, 1978). These studies provided key insights into the mystery of the hypothalamic-pituitary-adrenal (HPA) axis—a pathway critical for the physiological response to stress.

Research in subsequent decades from Wylie Vale on corticotropic releasing hormone and many others better solidified how stress operates across the brain and body (Spiess et al., 1981). Indeed, Vale's work was complimented by pioneering studies in the 1980s and 1990s from Bruce McEwen as well as Jim McGaugh on how cortisol can enter the brain and bind to corticoid receptors to influence several cognitive processes (e.g., allostatic load, etc.) including memory (McEwen and Sapolsky, 1995; McGaugh, 2000). In the intervening and subsequent years, many other scientists detailed how different cognitive processes and molecules within the brain are influenced by stress (for review see Joëls and Baram, 2009).

In recent decades, we have made significant progress toward identifying how stressors across physical, individual, temporal, and social domains, impact both the brain and body (McEwen, 2007). However, there is still a considerable road ahead toward identifying how neuronal circuits of the brain adapt to and regulate our response to stressors over time. The need to identify which circuits, cells, and molecules mediate risk and resilience is vital to developing new treatments—with applications ranging from post-traumatic stress disorder to anxiety.

In this Research Topic, we collected reviews and research ranging from theoretical to experimental with insights across the lifespan. We first highlight work in a review by Rosen and Schulkin at the University of Delaware leverages older ideas of kindling (often related to seizures) from the 1980s to describe how excitability states in neuronal circuits of the hippocampus and amygdala—brain regions critical for stress, fear, and memory—may provide a framework for understanding mental health disorders related to post-traumatic stress disorder (PTSD). Second, work by Packard et al. in Regina Sullivan's group at New York University leverages a tremendous history dating back to the seminal work of Harry Harlow on how mother-infant attachment styles interact with corticosterone across the hippocampus and amygdala to alter social behavior. Third, work from Love in Zelikowsky's group at the University of Utah expand on ideas of early social bonds to better frame how different social factors impact stress and mental-health outcomes (Love and Zelikowsky). Finally, Laura Graffe from Seema Bhatnagar's group at the University of Pennsylvania provides original research on how the impact of social stress extends beyond physiological changes to impact patterns of sleep with relevance to PTSD (Grafe et al.). Taken together, these papers highlight how stress reaches across a variety of physiological and psychological domains. Research in the coming years will provide critical insights into how stress across the lifespan impacts neuronal circuits as well as the molecular biology of risk and resiliency, with the goal of developing better treatments to help the millions who suffer from stress-related disorders.

## Author contributions

The author confirms being the sole contributor of this work and has approved it for publication.

## Conflict of interest

Author AA was employed by Alien Therapeutics Inc.

## Publisher's note

All claims expressed in this article are solely those of the authors and do not necessarily represent those of their affiliated organizations, or those of the publisher, the editors and the reviewers. Any product that may be evaluated in this article, or claim that may be made by its manufacturer, is not guaranteed or endorsed by the publisher.
